# Legionnaires' Disease Complicated with Rhabdomyolysis and Acute Kidney Injury in an AIDS Patient

**DOI:** 10.1155/2017/8051096

**Published:** 2017-10-04

**Authors:** Karan Seegobin, Satish Maharaj, Cherisse Baldeo, Julio Perez Downes, Pramod Reddy

**Affiliations:** Department of Internal Medicine, University of Florida College of Medicine, Jacksonville, FL 32209, USA

## Abstract

**Objective:**

To present a case of an uncommon triad of* Legionella* pneumonia, rhabdomyolysis, and renal failure, with review of the relevant literature.

**Case:**

A 51-year-old with a history of human immunodeficiency virus (HIV), chronic obstructive pulmonary disease (COPD), and hypertension presented with fever, cough, and shortness of breath over four days. Chest X-ray showed consolidation in left lower lung field; urine was positive for Legionella antigen and myoglobin; creatine kinase was 51092U/L; creatine was 6.9 mg/dL, and his CD4 count was 41 cells/ul. He was managed with azithromycin and levofloxacin and further required dialysis and ventilatory support in the intensive care unit due to renal failure and respiratory failure. He responded well to the treatment and made a complete recovery.* Legionella pneumophila* infection is a recognized but rare cause of rhabdomyolysis with high morbidity and mortality when there is extrapulmonary involvement. Early diagnosis and appropriate treatment is essential to improve outcomes.

**Conclusion:**

Physicians should consider* Legionella* pneumonia in patients with rhabdomyolysis, renal failure, and respiratory symptoms. Early diagnosis and treatment have been shown to have good clinical response. Timely intensive care management, together with early and judicious use of dialysis in patients complicated with rhabdomyolysis and renal failure, may lead to good outcomes.

## 1. Introduction

*Legionella pneumophila* (LP) infection is a recognized but rare cause of rhabdomyolysis [1, 2]. It can be further complicated with renal impairment which can be due to acute tubular necrosis (ATN) or acute tubulointerstitial nephritis (ATIN) [3]. This triad of pneumonia, renal failure, and rhabdomyolysis is associated with high morbidity and mortality [4]. We report a case of an immunocompromised patient with* Legionella* pneumonia complicated with acute kidney injury and rhabdomyolysis. He required dual antibiotics with levofloxacin and azithromycin, in addition to dialysis, and intensive care treatment. He made a full recovery with normal renal function. After review of the relevant literature on reported cases of this uncommon triad of* Legionella* pneumonia, renal failure, and rhabdomyolysis, early diagnosis has been shown to have good clinical response. We advocate for timely transfer to the intensive care unit (ICU) and judicious use of dialysis in patients with complicated* Legionella pneumophilia* as the outcomes are good.

## 2. Case

A 51-year-old male with a past medical history of HIV, COPD, and hypertension presented with a four-day history of fever, shortness of breath, and nonproductive cough associated with headache and reduced appetite. He had a 30-pack-year history of smoking cigarettes and had not been compliant with his HIV medications as well as trimethoprim-sulfamethoxazole. He had no history of nonsteroidal anti-inflammatory drug (NSAID), herbal drug, or cocaine use. He denied recent ill contacts, recent travel, or camping. On examination, he was in respiratory distress with blood pressure 143/95 mmHg, pulse 135 beats per minute, respiratory rate 24 breaths per minute, temperature 39.5 degrees Celsius, and sPO2 92% on room air and 98% on 2 litres nasal cannula. He had bronchial breath sounds in the left mid and lower lung fields with crackles, but no wheezing. His heart sounds were normal. Abdomen was soft and nontender, with normal bowel sounds. Other aspects of his examination were unremarkable.

His white cell count was 4.6 (4.5–11 × 10^3^/uL); haemoglobin was 10.7 (12–16 g/dL); platelet was 246 (140–440 thou/cu mm); mean corpuscular volume was 94 (82–101 fl); Prothrombin Time (PT) was 12.9 (11–13.5 seconds); international normalised ratio (INR) was 1.0 (0.8–1.2); partial thromboplastin time (PTT) was 34 (25–35 seconds); HbA1c was 5.7% (4–5.6%); procalcitonin was 31.2 ng/mL (<0.15 ng/mL); thyroid stimulating hormone (TSH) was 0.705 (0.27–4.2 uIU·ml); sodium was 137 (135–145 mmol/L); potassium was 4.5 (3.3–4.6 mmol/L); chloride was 95 (101–110 mmol/L); BUN was 56 (6–22 mg/dL); creatine was 6.98 (0.6–1.17 mg/dL); calcium was 7.8 (8.6–10 mg/dL); albumin was 2.6 (3.8–4.9 g/dL); phosphorus was 5.5 (2.5–4.5 mg/dL); aspartate aminotransferase was 789 (14–33 IU/L); ALT was 235 (10–42 IU/L); anion gap was 22 (4–16); direct bilirubin was 3.3 (0–0.2 mg/dL); indirect bilirubin was 0.4 mg/dL (0.2–1.2 mg/dL); total bilirubin was 3.7 (0.2–1.0 mg/dL); alkaline phosphatase was 66 (40–129 IU/L); total creatine kinase was 51092 U/L (22–195 U/L), and urine was positive for myoglobin. His CD 4 count was 41 cells/ul, and HIV viral load was 34900 copies/mL.

Urine analysis with Alere BinaxNOW lateral flow immunochromatographic assay for* Legionella* antigen serogroup 1 was positive; urine pneumococcal antigen was negative; blood culture, respiratory culture, and urine culture were without bacterial growth; respiratory viral panel was reported negative for influenza, parainfluenza, rhinovirus, respiratory syncytial virus (RSV) virus, human metapneumovirus, H1, and H3.

**Figure 1:**
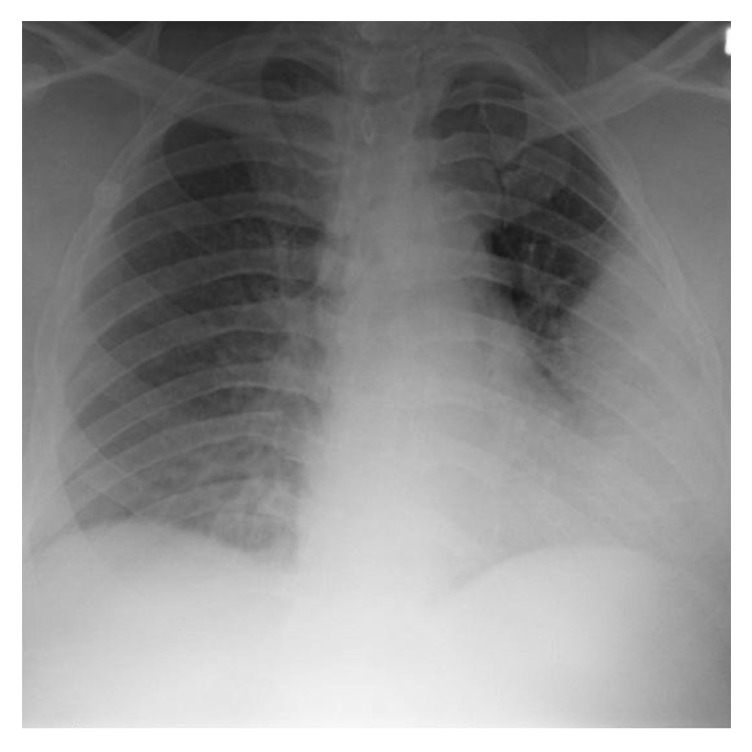
Chest X-ray with homogenous consolidation in the left lower lung field.

**Figure 2:**
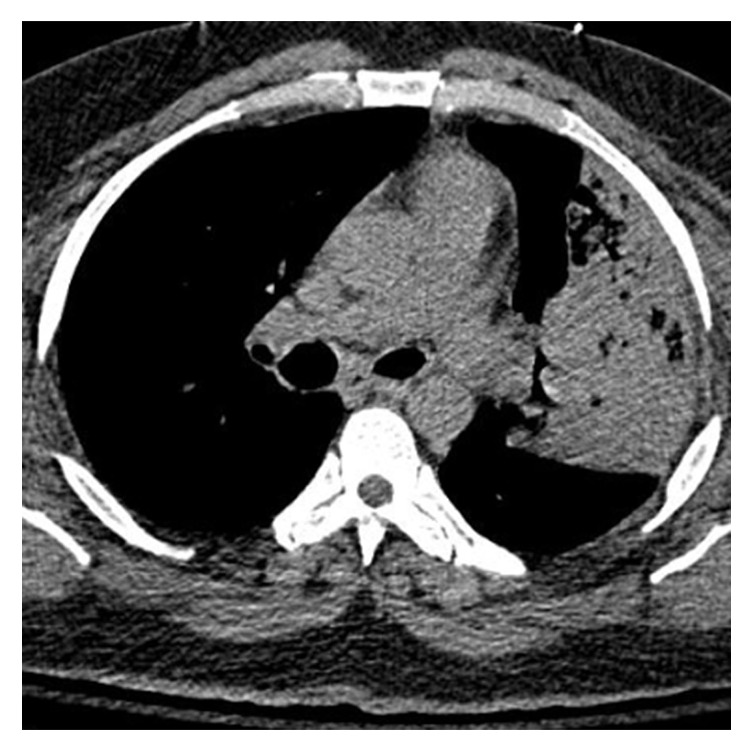
Axial section chest CT showing consolidation in the left lingual with air bronchograms consistent with lobar pneumonia, without pleural effusion.

**Figure 3:**
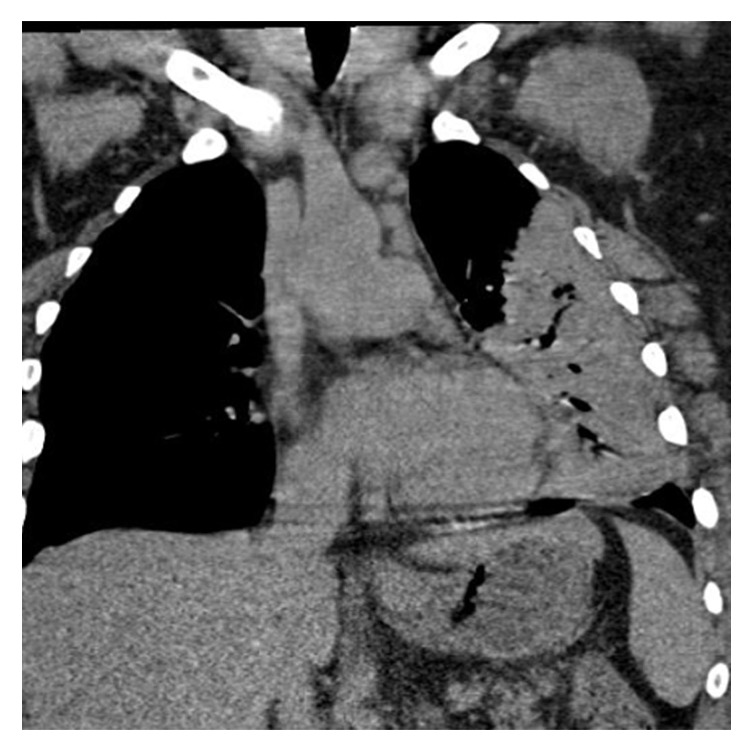
Coronal section chest CT showing consolidation in the left midzone with air bronchograms consistent with lobar pneumonia, without pleural effusion.

His electrocardiogram showed sinus tachycardia. Chest X-ray (CXR) showed a homogenous consolidation in the left lower lung field (Figure 1). CT chest showed consolidation in the left lingual with air bronchograms consistent with lobar pneumonia, without pleural effusion (Figures 2 and 3). Echocardiograph showed a left ventricular ejection fraction of 65%. Renal ultrasound showed normal sized kidneys with normal echogenicity without hydronephrosis.

**Figure 4:**
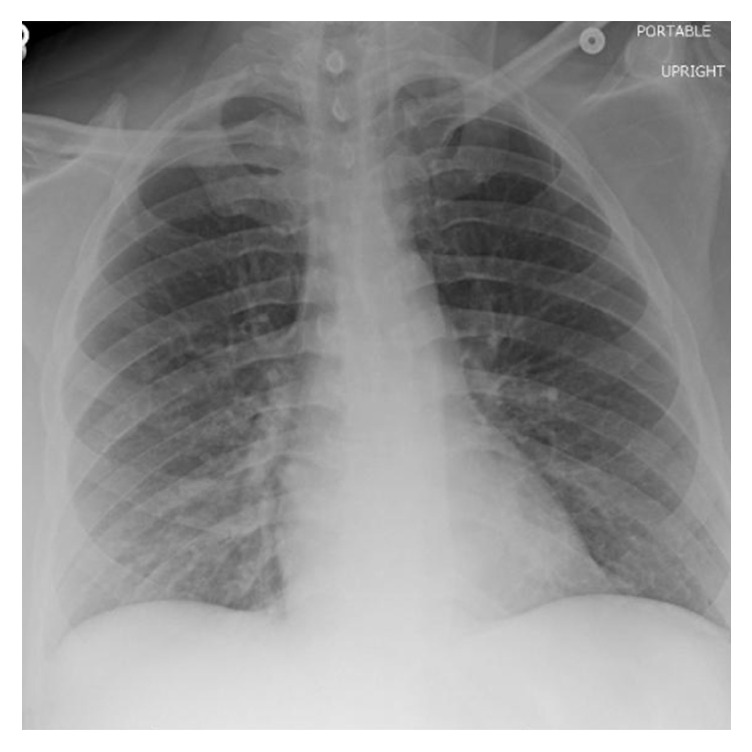
CXR after three-week follow-up, without consolidation.

He was diagnosed with* Legionella* pneumonia, rhabdomyolysis, and acute renal failure and started on levofloxacin and atovaquone. He became further oliguric, with rising creatine and BUN, with worsening respiratory status requiring intubation and ventilation in addition to dialysis which were commenced on the second day of admission. Azithromycin was added to his antibiotic regimen in the ICU. His maximum creatine was 13.04 mg/dL, and BUN was 153 mg/dL during his hospital course. His respiratory and renal function improved during his ICU stay. He was extubated after six days. With improvement in his urine output and renal indices; he no longer required dialysis. On discharge, after 28 days of hospital stay and seven days of ICU stay, his creatine was 2.6 md/dL, BUN 36 md/dL, total creatine kinase (CK) 69 U/L, aspartate aminotransferase (AST) 16 IU/L, alanine aminotransferase (ALT) 30 IU/L, direct bilirubin 0.1 mg/dL, indirect bilirubin 0.1 mg/dL, and total bilirubin 0.2 mg/dL. After three-weeks follow-up in the outpatient setting, his creatine was back to normal at 1.06 (0.6–1.17 mg/dL), with resolution of the chest X-ray consolidation (Figure 4). Renal biopsy was not pursued in light of recovery of normal renal function.

## 3. Discussion

In adults,* Legionella* causes 2–15% of community acquired pneumonia (CAP) cases that require hospitalization [5]. It is the second most common cause of serious pneumonia that needs admission in an intensive care unit (ICU) [5]. The first report that associated* Legionella* and rhabdomyolysis was published in 1980 by Posner et al. [5] It is a recognized but rare cause of rhabdomyolysis [1, 2]; a clinical syndrome characterized by elevated serum concentrations of creatine phosphokinase (CPK) and myoglobinuria leading to renal dysfunction [17].

Renal impairment in* Legionella pneumophila *infection accompanied by rhabdomyolysis can be due to ATN or ATIN [3].* Legionellosis*-associated ATIN could be either indirectly associated with* L pneumophila* via rhabdomyolysis or directly affected by* L pneumophila *[3]. Interestingly, renal anomalies can develop days before the imaging demonstration of pneumonia [18].

Many of the laboratory findings are nonspecific and include renal and hepatic dysfunction, hyponatremia, hypophosphatemia, thrombocytopenia, leukocytosis, hematuria, and proteinuria [1]. The gold standard is culture and sensitivity on specialized charcoal media, which has a sensitivity of 70–80% [1]. However, this is not a rapid test and usually takes 3–5 days [1]. Urine antigen test is a rapid, practical, and inexpensive method for the diagnosis of the disease, characterized by sensitivities of 70–90% and specificities approaching 100% [4, 5]. In our case the use of urine antigen testing confirmed the etiology of the pneumonia.

From a search on PubMed and Google with the terms “*Legionella*”, “rhabdomyolysis”, and “renal failure” we analysed case reports (Table 1) of 16 patients with the triad of renal failure, rhabdomyolysis, and* Legionella* pneumonia with focus on CK levels, need for dialysis, intensive care admission, outcomes after treatment, and the use of quinolones, macrolides, or both.

Thirteen patients made full recovery, five of which were treated with a quinolone and macrolide, with the others having either a quinolone or macrolide as part of their treatment. Observational studies suggest that quinolones are more likely to achieve a favourable outcome in terms of patient survival and length of hospitalization [5]. Combined treatment is believed to be superior to monotherapy in cases of severe clinical disease or in immunosuppressed subjects [5]. In our case early treatment with a quinolone and macrolide led to good outcome.

Of the 16 patients analysed, 8 patients had CK levels greater than 5,000 U/L at presentation, with four out of those 8 having levels >20,000 U/L. Our patient also had a significantly high CK level at 51092 U/L. Considering that patients present to the hospital days after the onset of disease, this may give the bacteria sufficient time to cause muscle injury. The exact mechanism of muscle injury caused by* Legionella* is still unclear [19]. However, release of an endotoxin or exotoxin that causes rhabdomyolysis and direct bacterial invasion seem to be the most probable mechanisms [19]. In another report published in 1992 that studied renal failure in patients with* Legionella *disease, 8 patients had rhabdomyolysis out of 45 cases, three of which died; 2 of which did not undergo dialysis [6]. Physicians should always anticipate rhabdomyolysis and check for its presence in patients with* Legionella* disease, as early anticipation of this event and initiation of early aggressive fluid resuscitation are critical in reducing the risk of myoglobinuric acute kidney injury [20]. CK levels have been shown to correlate well with severity of rhabdomyolysis in some reports [21]. Furthermore, in one study of 1,769 adult patients, serum CK > 773 U/L correlated well with the onset of acute kidney injury (AKI) and rhabdomyolysis [21]. Many of the patients from our analysis in addition to our patient had the presence of renal impairment with CK levels > 773 U/L [22].

From our analysis of 16 patients, most cases required ICU care (10 cases). A 1998 study of 392 cases of community acquired pneumonia revealed 12.5% due to* Legionella* and 20.4% of those requiring ICU admission [1]. It is reported that chronic lung disease, immunodeficiency, malignancies, renal impairment, diabetes mellitus, nosocomial acquisition, and delayed initiation of specific antimicrobial therapy are associated with poor outcomes [5]. Our patient had four of these factors which could have predisposed him to a more severe course of* Legionella *disease. Additionally, it is reported that late but not early admission to the ICU for community acquired pneumonia is associated with poorer outcomes in comparison to patients managed on medical wards [23]. From our analysis, of the 10 patients admitted to the ICU, seven had good outcomes. Our patient also had a good recovery. It is important that there be early identification of patients who may develop severe* Legionella* pneumonia within the very first days of hospital stay to intensify the level of care and to propose early ICU transfer as the outcomes may be better.

From this review of 16 patients, most of these patients became progressively oliguric with worsening renal indices during their hospital course eventually requiring dialysis as seen in 12 of these cases. Our patient took a similar course despite aggressive fluid resuscitation. With this trend, frequent monitoring of renal indices and urine output is critical in patients with* Legionella *disease in light of the propensity for this disease to follow the path of eventually requiring dialysis, as seen in our case. The timing of initiating dialysis is a challenge and should not be determined solely by myoglobin or CK serum concentration but by the status of renal impairment, with complications such as life-threatening hyperkalemia, hypercalcemia, hyperazotemia, anuria, or hyperhydration without response [21].

It is generally accepted that patients with immune paresis, especially cell-mediated, are susceptible to* Legionella *infection [24]. Nevertheless,* Legionella pneumophila* is not commonly described in persons with infection due to human immunodeficiency virus (HIV) [25]. There is no clear relation between incidence of* Legionella* CAP and CD4 count [26, 27]. In a 10-year retrospective single center study, 15 cases of* Legionella *CAP were observed. When compared with pneumococcal CAP, disease was more severe with* Legionella* with a higher incidence of bilateral pneumonia, respiratory failure, and need for ventilatory support. Extra respiratory symptoms, hyponatremia, and increased creatine phosphokinase were more frequent in* Legionella* pneumonia [27]. Our patient was not compliant with prophylactic trimethoprim-sulfamethoxazole (TMP-SMX). This drug has some activity against* Legionella *and in previous reports there is a low incidence of* Legionella* pneumonia in persons on TMP-SMX prophylaxis [25]. Health professionals should have high suspicion for* Legionella* as a possible etiology for pneumonia, in AIDS patients with TMP-SMX noncompliance.

## 4. Conclusion

Consider* Legionella* pneumonia in patients presenting with rhabdomyolysis, renal failure, and respiratory symptoms. Early diagnosis and appropriate treatment have been shown to have good clinical response. Timely intensive care management, together with early and judicious use of dialysis, in patients complicated with rhabdomyolysis and renal failure may lead to good outcomes.

## Figures and Tables

**Table 1:** 

Reference	Age (years)	Dialysis required	ICU treatment needed	Antimicrobial class (quinolone, macrolide, or both quinolone and macrolide)	Outcome
Koufakis et al. [5]	45	Yes	Yes	Quinolone and macrolide	Recovery
Shimura et al. [3]	54	Yes	Yes	Quinolone and macrolide	Recovery
McConkey et al. [1]	56	No	Yes	Quinolone	Recovery
Shah et al. [6]	26	Yes	Undetermined	Macrolide	Recovery
Erdogan et al. [4]	67	Yes	Undetermined	Quinolone and macrolide	Recovery
Abe et al. [7]	56	Yes	Yes	Quinolone and macrolide	Died
Wiegele and Krenn [8]	44	Yes	Yes	Undetermined	Recovery
Linga and Deo [2]	40	Undetermined	Yes	Undetermined	Recovery
Agu et al. [9]	45	No	Undetermined	Quinolone	Recovery
Nakatani et al. [10]	50	Yes	Undetermined	Quinolone	Recovery
Li et al. [11]	55	Yes	Undetermined	Quinolone and macrolide	Recovery
Daumas et al. [12]	55	Yes	Undetermined	Quinolone and macrolide	Recovery
Narita et al. [13]	48	Yes	Yes	Quinolone	Recovery
Sposato et al. [14]	61	Yes	Yes	Macrolide	Died
Matsumoto et al. [15]	67	Yes	Yes	Macrolide	Recovery
Tokuda et al. [16]	57	Undetermined	Yes	Macrolide	Died
